# Evaluation of effectiveness of class-based nutrition intervention on changes in soft drink and milk consumption among young adults

**DOI:** 10.1186/1475-2891-8-50

**Published:** 2009-10-26

**Authors:** Eun-Jeong Ha, Natalie Caine-Bish, Christopher Holloman, Karen Lowry-Gordon

**Affiliations:** 1 Family and Consumer Studies, Kent State University, 100 Nixson Hall, Kent, OH 44242, USA; 2 Department of Statistics, the Ohio State University, 1958 Neil Ave, Columbus, Oh 43210, USA

## Abstract

**Background:**

During last few decades, soft drink consumption has steadily increased while milk intake has decreased. Excess consumption of soft drinks and low milk intake may pose risks of several diseases such as dental caries, obesity, and osteoporosis. Although beverage consumption habits form during young adulthood, which has a strong impact on beverage choices in later life, nutrition education programs on beverages are scarce in this population. The purpose of this investigation was 1) to assess soft drink and milk consumption and 2) to evaluate the effectiveness of 15-week class-based nutrition intervention in changing beverage choices among college students.

**Methods:**

A total of 80 college students aged 18 to 24 years who were enrolled in basic nutrition class participated in the study. Three-day dietary records were collected, verified, and analyzed before and after the intervention. Class lectures focused on healthful dietary choices related to prevention of chronic diseases and were combined with interactive hands on activities and dietary feedback.

**Results:**

Class-based nutrition intervention combining traditional lecture and interactive activities was successful in decreasing soft drink consumption. Total milk consumption, specifically fat free milk, increased in females and male students changed milk choice favoring skim milk over low fat milk. (1% and 2%).

**Conclusion:**

Class-based nutrition education focusing on prevention of chronic diseases can be an effective strategy in improving both male and female college students' beverage choices. Using this type of intervention in a general nutrition course may be an effective approach to motivate changes in eating behaviors in a college setting.

## Background

In the USA, carbonated soft drinks and milk are the two most popular non-alcoholic beverages, accounting for 39.1% of total beverage consumption [1]. Soft drink consumption has exploded over the past three decades [2] demonstrating a per capita availability increase from 22 gallons to 52 gallons [3,4]. Sugar sweetened soft drinks became a major source of added sugar in the American diet [5,6] and have been linked to adverse nutritional and health consequences such as dental caries and obesity [5,7-12]. Furthermore, evidence also supports an association between soft drink consumption and decreased bone mineral density (BMD) [8,13,14].

Milk and other dairy products are the major source of dietary calcium contributing to about 70% of the calcium in the U.S. food supply [3]. Sixty years ago, Americans drank more than four times more milk as compared to soft drinks, but 2 1/3 times more soft drinks were consumed than milk by 1998 [3]. This trend demonstrates a possible displacement of milk intake [15]. In addition, data showed that between age 6 and 19 years, age is positively associated with soft drink consumption and negatively with milk intake [16]. This relationship is most prevalent in adolescents and young adults [13]. Sufficient intake of calcium, especially during adolescence and young adulthood, is important to maximize peak bone mass (PBM). Failure to achieve PBM increases the incidence of osteoporotic fracture later in life [17].

Young adulthood is a unique period whereby youth obtain independence from their parents. People in this age group are vulnerable to develop unhealthy behaviors [18,19], which will predispose them to chronic diseases later in life [20]. A longitudinal study tracking soft drink intake from early adolescence to later adulthood demonstrated that soft drink consumption from young adulthood remained stable [21]. This data indicates beverage consumption habits formed during young adulthood may have a strong impact on beverage choices in later life. In addition, since milk intake decreases with age after childhood, there is an urgent need for tailored nutrition intervention targeting the young adults to improve their beverage choices.

The purpose of this investigation was two-fold: 1. to assess soft drink and milk consumption and 2. to evaluate the effectiveness of 15-week class-based nutrition intervention in changing beverage choices among college students.

## Methods

During spring 2006, ninety healthy college students, between the ages of 18 and 24 years, enrolled in a basic sophomore level nutrition class at a Midwest University participated in the study. This research was approved by the University Institutional Review Board and informed consent was obtained from each participant before enrollment in the project.

The present study used a pre-post test design. Data were collected during the first two weeks and the last week of spring semester in 2006. Body weight was measured in kilograms to the nearest 0.1 kilogram on an electronic scale in light clothing without shoes. Standing height was recorded without shoes on a portable stadiometer to the nearest 0.1 centimeter with mandible plane parallel to the floor. Each subject's BMI was calculated as weight (kg)/height ^2^(m).

Dietary intake was assessed using 3-day dietary records for two typical weekdays and one weekend day. A variety of tools were used to obtain reliable data. Food models, measuring cups and spoons, household utensils, and tableware were used to illustrate proper portion sizes. Participants were asked to collect and bring all the food labels of products they consumed during data collection period. To obtain the most accurate dietary data, research associates visited local restaurants and campus cafeterias where the majority of participants ate to gain accurate information about ingredients and portion sizes. Foods were purchased if needed. Dietary analysis was performed by the same individual using NutriBase IV Clinical (Cyber Soft Inc, Arizona).

The class met three times per week for 50 minutes per session. Class lectures specifically emphasized 1) the importance of nutrition related to prevention of chronic diseases, 2) increasing consumption of fruits, vegetable and whole grain products, 3) encouraging low fat dairy product consumption, 4) discouraging over reliance on dietary supplements and 5) promoting active lifestyle. In addition to the traditional approach by lectures, video-tape watching and various hands-on activities were integrated. Hands-on activities were designed to enable students to translate lecture materials into real life application. For example, after lectures of lipid and calcium, students assessed their risks for heart disease and osteoporosis, by completing risk assessment forms. These activities helped students identify risk factors and realize that they are not free of chronic disease risks just because they are young or currently disease free. In addition, students completed "Happy Body Log" and listed good things that they did for their body in a daily log. The key of this activity was to start with small behavior changes such as: not eating while watching T.V., reducing portions of single condiments, choosing skim milk over 2% milk. Another approach to encourage dietary behavior change included returning the results of dietary analysis to the students. They were asked to bring their returned results to every class. During lectures, students compared their actual intakes to dietary recommendations (i.e. MyPyramid and Dietary Recommended Intake), which allowed them to realize the strengths and weaknesses of their diet.

Descriptive statistics were presented as means and standard errors. Repeated measures ANOVA with gender as a between-subjects factor and time as a within-subjects factor was used to compare consumption of total soft drink, regular soft drink, diet soft drink, total milk, low fat milk, and fat free milk before and after the intervention. Because there were many more females than males in the class, paired t-tests would be heavily biased toward the females. Therefore, the estimated marginal means obtained from a repeated measures linear model were provided for the pre- and post-test, weighting males and females equally. Estimated marginal means were chosen to represent the total effect since the population of interest includes all college students. Among college students, males and females are represented more evenly than in our sample, so such a summary is justified. Significance tests for the marginal means draw power from the full sample, while the effect size is a compromise between the effect sizes for males and females. As a result, it is possible to have non-significant effects for each gender alone, but significant effects for the marginal means. In addition to the values in the table, total calcium intake and calcium intake from milk at pre- and posttest were calculated. Spearman correlations were calculated to quantify correlations among variables before and after the intervention. Correlations were calculated among change scores. Significance was set a priori at P ≤ 0.05. All analyses were performed using SPSS for Windows (version 15.5, 2007, SPSS, Chicago, Ill). The result of this study is limited to beverage consumption and the results from other data from the food records have been published elsewhere [22].

## Results

Among ninety students enrolled in a sophomore level general nutrition course, 80 students completed the study. Participants were mainly females (87.5%) and white (89.7%). Average BMI of the participants was 26.3 ± 5.63 kg/m^2^. Average age of the participants was 20.15 ± 1.38 years.

Table 1 summarizes the data and statistical tests on change in beverage consumption as a result of the intervention.

**Table 1:** Pre- & Posttest Daily Intake of Beverage by Gender (Means ± Standard Errors, Repeated Measures Analysis)

	**Gender (n)**	**Pretest (fl.oz) Mean (SE)**	**Posttest (fl.oz) Mean (SE)**	**p-value**
Total soft drink				
	Male (9)	8.53 (3.29)	4.74 (2.27)	0.093
	Female (70)	4.94 (0.84)	3.62 (0.62)	0.100
	Estimated Marginal Mean	6.73 (1.30)	4.18 (0.95)	0.033*

Regular soft drink				
	Male (8)	5.33 (3.36)	2.96 (2.28)	0.145
	Female (70)	2.11 (0.45)	1.28 (0.41)	0.072
	Estimated Marginal Mean	3.72 (0.86)	2.30 (0.72)	0.051

Diet soft drink				
	Male (9)	3.79 (2.31)	1.78 (1.35)	0.346
	Female (70)	2.83 (0.74)	2.30 (0.54)	0.490
	Estimated Marginal Mean	3.31 (1.11)	2.04 (0.79)	0.263

Total milk				
	Male (9)	6.62 (2.32)	6.63 (2.41)	0.997
	Female (70)	4.18 (0.71)	6.23 (0.85)	0.022*
	Estimated Marginal Mean	5.40 (1.07)	6.43 (1.26)	0.433

Whole milk				
	Male (9)	0.00	0.00	1.000
	Female (69)	0.58 (0.31)	0.13 (0.13)	0.149
	Estimated Marginal Mean	0.29 (0.44)	0.07 (0.19)	0.621

Low fat milk				
	Male (9)	6.18 (2.42)	1.70 (1.11)	0.020*
	Female (69)	2.16 (0.52)	2.09 (0.54)	0.918
	Estimated Marginal Mean	4.17 (0.84)	1.90 (0.77)	0.027*

Fat free milk				
	Male (9)	0.44 (1.33)	4.93 (7.75)	0.052
	Female (69)	1.67 (0.51)	3.54 (0.78)	0.026*
	Estimated Marginal Mean	1.06 (0.71)	4.23 (1.18)	0.010*

*demonstrates significant difference P ≤ 0.05

Total soft drink consumption significantly decreased from baseline (P < 0.05), although there was insufficient evidence to declare a significant difference for either gender alone. There was marginal evidence that regular soft drink consumption at posttest decreased from the baseline. No change in the consumption of diet soft drink was demonstrated.

For total milk, combining results across genders, no significant change was observed. However, the average change in total milk consumption was significantly increased from baseline (P < 0.05) for females but not for males. Whole milk consumption at baseline did not change after the intervention in either gender. Low fat milk consumption decreased significantly (P < 0.05) due to a significant change in the males' consumption patterns whereby, there was a significant increase in fat free milk intake after the intervention (P < 0.01). This effect was observed to be significant in females (P < 0.05) and marginally significant in males.

Total calcium intake at pretest was 813.18 ± 501.48 mg and 858.21 ± 373.11 mg at posttest, respectively. Calcium intake contributed by milk consumption was 156.75 mg at the pretest and 233.0 mg after the intervention.

Correlation coefficients between milk and soft drink consumption were not significant at baseline and remained unchanged after the intervention. In addition, changes in consumption for each type of drink were not correlated with each other except for an observed negative correlation between the change in fat free milk intake and the change in low fat milk consumption whereby as fat free milk consumption increased low fat milk consumption decreased (r = -0.317, P < 0.05). In addition, there was a positive correlation between milk consumption and dietary calcium intake (r = 0.578, P < 0.001) at baseline, which further increased after the intervention (r = .689, P < 0.001).

## Discussion

The results of this study provided evidence that class-based nutrition education was a viable mechanism to use to help college students make positive changes in soft drink and milk consumption. Previous literature has demonstrated that there have been several studies using college nutrition courses to motivate overall dietary changes [23,24]. Results of this research indicated that nutrition courses increased nutrition knowledge but did not promote dietary changes. On the other hand, a study using a college nutrition science course to prevent weight gain in freshmen revealed that class-based nutrition education may help college students translate nutrition knowledge into dietary changes [25]. Overall, prior research on interventions targeting college students' dietary behaviors suggest a need to develop curriculums targeting specific nutrition behaviors in college students

After the intervention, overall total soft drink consumption had significantly decreased from baseline. The decrease in total soft drink consumption was mainly due to the reduction in regular soft drink consumption because diet soft drink intake did not decrease as a result of the intervention. The general nutrition class designed to increase the awareness of importance of nutrition in prevention of chronic disease through the combination of traditional lecture with interactive activities may have encouraged the students to reduce soft drink consumption as a part of healthy eating practices. Although it is still debated whether soft drink consumption is associated with increasing obesity rates or decreased milk consumption, it is evident that soft drink consumption has been linked to some negative life style and dietary patterns [26-29]. In a cluster study, Kvaavik et al. found that soft drink consumption could be a marker of unhealthy eating behaviors [16] indicating that reduced intake of soft drink in the current investigation may reflect increased overall diet quality by class-based nutrition intervention.

It should be noted that the amount of soft drinks consumed before the intervention was lower than the results reported by other researchers [30,31] who reported daily soft drink intake of young adults between 11 and 14.4 ounces. There are several reasons to explain this discrepancy. In a study of adolescents, Bere et al. [32] reported that the participants who planned to receive college education showed lower odds of drinking soft drink. Cullen et al. [33] also found that lower parental education was associated with higher consumption of soft drinks. This data perhaps suggests that lower soft drink consumption in the current study may have been due to the higher education level of the participants, college students, compared to the study population, a mixture of both college students and young adults not enrolled in college, used in the previous studies [31].

A second positive finding of this study is that, although total milk consumption did not increase significantly between the genders, females increased their total milk consumption by increasing fat free milk intake while maintaining their low fat milk intake at the same level. Daily calcium intake contributed by milk consumption in females was 156.75 mg at the pretest and 233.0 mg after the intervention. This indicates that only 19% of total calcium intake was coming from milk before intervention and 25% after intervention. This is an encouraging finding because females are at an increased risk to develop osteoporosis in later life if calcium intake is compromised during adolescence and young adulthood. Meanwhile, males switched their milk choices from low fat milk to fat free milk since their total milk consumption did not change, which may demonstrate males may not recognize osteoporosis as an immediate danger due to a broad notion that osteoporosis an "old woman's disease" [28]. It may be that the males chose fat free milk over low fat milk in an attempt to reduce fat intake, which was an important educational component in the classroom lectures and projects. However, it should be noted that, even after the intervention, milk and calcium intake was still much lower than the recommended levels, 3 cups per day by MyPyramid [34] or 1000 mg [35] for both genders at pre- or post-test, although total milk consumption increased in females after the intervention. This finding underscores the necessity of nutrition intervention specifically designed to increase calcium intake in college students.

The positive correlation between dietary calcium and milk intake supports the idea that increasing milk consumption is a desirable way to encourage calcium intake to promote adequate bone health.

Over the last two decades, several researchers have reported that a reduction in milk intake coincides with an increased consumption of soft drinks consumption and hypothesized that soft drink has displaced milk [6,15]. However, in agreement with the previous finding by Storey et al. [36], the current study revealed no association between soft drink consumption and milk intake at either baseline or posttest, perhaps suggesting that soft drink consumption did not displace milk consumption in this population. This finding may imply that educating individuals to decrease soft drink consumption is not going to directing increase dairy consumption and that further dairy education needs to be addressed to ensure an adequate consumption of dairy products other than milk.

A limitation of this study is that a convenience sample without a control group was used. Therefore, the study population may not represent traditional college students. In addition, possible confounding factors, such as seasonal variation in beverage consumption, were not controlled for.

In conclusion, class based nutrition education intervention which focused on the prevention of chronic diseases has the potential in college students to reduce soft drink consumption and to increase milk consumption, specifically fat free milk, in female students and to alter milk choice in males from low fat milk to skim milk. Using this type of intervention in a general nutrition course may be an effective approach to motivate changes in eating behaviors in a college setting. Considering gender differences in changes in milk intake, future intervention programs may require different strategies for males emphasizing osteoporosis risk in men and the importance of osteoporosis prevention at earlier stages of life.

## Competing interests

The authors declare that they have no competing interests.

## Authors' contributions

EH designed the study. EH and NC were responsible for data collection. EH and CH conducted data analysis. EH, NC, CH and KL interpreted the analysis contributed to writing and revising the manuscript. All authors read and approved the final manuscript.
